# Outcomes of AV fistula formation for dialysis patients using interrupted sutures one-year single center study

**DOI:** 10.12669/pjms.40.5.8990

**Published:** 2024

**Authors:** Farah Naz, Rana Hassan Javaid, Sara Noor, Hira Katpar

**Affiliations:** 1 Dr. Farah Naz, MBBS. Plastic Surgery Resident, Department of Plastic Surgery, PNS Shifa Hospital, Karachi, Pakistan; 2 Dr. Rana Hassan Javaid, FRCS(Plast), FCPS(Plast), FCPS(G.S). HOD, Plastic Surgery, Professor of Surgery, BUMDC, Karachi, Pakistan; 3 Dr. Sara Noor, MBBS. Plastic Surgery Resident, Department of Plastic Surgery, PNS Shifa Hospital, Karachi, Pakistan; 4 Dr. Hira Katpar, MBBS. Plastic Surgery Resident, Department of Plastic Surgery, PNS Shifa Hospital, Karachi, Pakistan

**Keywords:** Outcomes, AVF, Dialysis, Interrupted sutures technique

## Abstract

**Objectives:**

To document the Outcomes of AV fistula formation for dialysis patients using interrupted sutures.

**Methods:**

In this Cross-sectional study conducted at PNS Shifa Karachi, from June 2022 to June 2023, patients above 18 years of age, male or female, with ESRD/CKD were included. After clinical screening and ultrasound doppler, the distal most part of wrist was considered as a site for radio cephalic AVF. Operation was performed under surgical loupe with 4.5x magnification. Standard incision of about 4-5 cm oblique proximal to the wrist crease was given at the volar surface on the radial side of Distal forearm, extending till the lateral side up to the snuffbox. Subcutaneous tissue was incised and dissection was done to identify the cephalic vein and radial artery. Distal most end of the cephalic vein was ligated using vicryl 4-0 suture and cephalic vein dissected free from the underling tissue to mobilize it up to the radial artery.

**Results:**

During the study N=35 patients who required AVF creation visited the department. The mean age of the study participants was 59.34±15.48. If thrill at the site of AVF and backflow at cut end of cephalic vein were present the surgeons were satisfied, higher satisfaction among the surgeon was achieved in the AVF created at brachiocephalic artery while the diameter of vessels didn’t contribute in surgeon satisfaction.

**Conclusion:**

Presence of thrill at the site of AVF and backflow at cut end of cephalic vein have strong association with good prognosis of AVF.

## INTRODUCTION

For over the two million people who require hemodialysis each year arteriovenous fistulae (AVF) remains the method to gain the permeant vascular access, it has been recognized as the primary and best option.^1^ When compared to prosthetic arteriovenous grafts (AVG) and central vein catheters (CVC), AVF have low infection rates, fewer chances of rehospitalization to revise the access, and lower chances of thrombus formation. Furthermore, it has been documented that patients with an AVF have significantly reduced mortality rates and longer life expectancies^2^. For an AVF a peripheral fistula is created first, wrist fistula (Brescia-Cimino AVF), a mid-forearm cephalic fistula, and finally a proximal forearm brachiocephalic fistula in the upper third of the forearm. However, creating AVF at the wrist level is the most common and preferred method to gain access.^3^

It has been documented that thrombus formation and failure to mature (FTM) rates pose a barrier to the development of a functional AVF. In European and American hemodialysis patients FTM rates were reported to be 24% and 60% respectively.^4^ The old age, diabetes mellitus, and nephrosclerosis are considered as the three main causes of ESRD, and these are also considered as the attributable factors for FTM rates.^5^ Additionally, in Pakistan, a study reported that the patency of an AVF depends on vessel diameter and arterial pressure hence assessment of these factors to predict the outcome of AVF.^6^ Despite having high failure rates wrist AVF has been suggested as the first and best hemodialysis access in American and European standards. The wrist AVFs had a lower incidence of steal syndromes, high-output cardiac failure, and edema related to central venous obstruction than brachial fossa fistulas do and it permits the maintenance of the patient’s vascular network and requires few treatments after maturation (infection, stenosis, and thrombosis).^5^

Furthermore, in an effort to improve flow dynamics and reduce early AVF failure, various operative alterations, such as side-to-side anastomoses, vein cuffs, and adjustments in the anastomotic angle, have been proposed. All have utilized conventional continuous suturing methods, although none of these have shown good results. Contrary to this, with excellent results, interrupted suturing techniques (or variations of interrupted suturing techniques with interrupted sutures for at least part of the anastomosis) are used in other clinical fields, such as free flap transfer, coronary artery bypass grafts, hepatic artery reconstruction, and small animal models, for microsurgical anastomoses (under an operating microscope or with surgical loupes). Improvements in anastomotic compliance, as well as a decrease in puckering and luminal narrowing, are all theoretical advantages. Therefore, assessing the outcomes after AVF formation may improve the quality of life of the patients hence, the study aims to document the Outcomes of AV fistula formation for dialysis patients using interrupted sutures.

## METHODS

It was a cross sectional study conducted at PNS Shifa Karachi. Patients who required AVF formation from June 2022 to June 2023 were included in the study.

### Ethical Approval:

The study was approved by the institutional review board and 26A approval number was allotted. (Date: June 30^th^ 2022.)

### Inclusion criteria:

- Patients above 18 years of age.
- Male or female.
- With ESRD/CKD stage.

### Exclusion criteria:

- Patients with gross edema of the non-dominant upper limb.
- Patients on blood thinners that could not be stopped due to cardiac reasons.
- Patients who did not consent for surgery.

### Preoperative preparation of patients:

Patients with CRF/ESRD were referred from nephrology department with the needs of permanent dialysis to the plastic and reconstructive surgery clinics for evaluation, as the institution did not have a vascular surgeon available.

Before surgery, the overall condition of the patients was assessed, history of recent vascular access and/or phlebotomy of cephalic veins was obtained. Patients were asked to not get any vascular access at the site where AVF was to be formed for up to three weeks. Patients with contraindications were excluded, physical examination of the patency of relevant veins and any bruising near the AVF sites was noted and Allen’s test was done.

### Procedure:

All procedures were performed by a single plastic surgeon. After clinical screening and ultrasound doppler, the distal most part of wrist was considered as a site for radio cephalic AVF. In other patients’ cubital fossa was also used for brachiocephalic AVF. Operation was performed under surgical loupe with 4.5x magnification. Standard incision of about four to five cm oblique proximal to the wrist crease was given at the volar surface on the radial side of Distal forearm, extending till the lateral side up to the snuffbox. Subcutaneous tissue was incised and dissection was done to identify the cephalic vein and radial artery. Distal most end of the cephalic vein was ligated using vicryl 4-0 suture and cephalic vein dissected free from the underling tissue to mobilize it up to the radial artery.

After dissecting the radial artery free from the underling structures, small tributaries were ligated. Two non-crushing clamps were applied on proximal and distal ends of radial artery. And one non-crushing soft clamp applied over the loose end of vein. Vertical arteriotomy was done in the radial artery, end to side anastomosis between artery and vein done taking small bites. using interrupted sutures with proline 8-0 sutures. Posterior wall was sutured first and then the anterior wall. All clamps were unsecured one-by-one and anastomosis checked for any bleeding and corrected. Once an easily palpable thrill was established, wound was closed with proline 4.0 sutures. Similar operative procedures were carried out for brachiocephalic AVF, the veins of choice were cephalic vein or median antecubital veins, and the artery was brachial artery. Usually, the sutures used for these anastomoses are 7-0 proline. Soft, padded dressing was done. Patients were asked to keep hand elevated and do not carry weight till the removal of sutures. First change of dressing was done on fifth post-operative day. Patients were called for follow-up for removal of sutures and then after 4-week interval to check for the maturation of fistula.

### Data collection and analysis:

Data was collected on a preformed proforma, during surgery backflow in cephalic vein after ligation and division, diameter of cephalic vein post anastomosis, thrill present right after anastomosis, pulse present after anastomosis, surgeon satisfaction post-surgery and thrill present post maturation of AV fistula were recorded. Data was analyzed by using SPSS version 22, independent sample t test was applied for comparison of vessel diameter after AVF in terms of surgeon satisfaction, chi square was used to associate the qualitative variables. The data analysis was performed at 95% confidence interval and p-value less then 0.05 was considered as significant.

**Fig.1:**
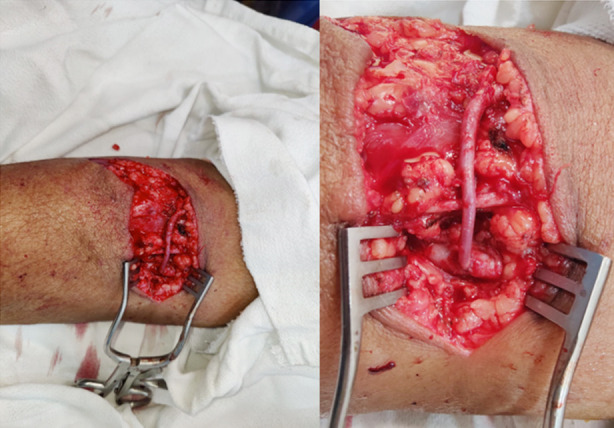
AVF fistula creation using interrupted suturing technique.

## RESULTS

During the study duration we received N=35 patients who required AVF creation for the dialysis. Among them 26 (74.3%) were males and 9 (25.7%) were females. The mean age of the study participants was 59.34 ± 15.48, the most common reason for AVF was ESRD followed by CKD. The data of co-morbidities among study participants is shown in Table-I. Among the study participants only one (2.9%) patient had non-function AVF, which was supposed to be redone at different site. Dopplers ultrasound was done prior to AVF formation and end to side anastomosis was created. In most of the patients AVF was created in Brachiocephalic artery 21 (60%) followed by radiocephalic artery 14 (40%). The outcomes of AVF after surgery is shown in Table-II.

**Table-I:** Comorbidities among the patients admitted for AVF.

Comorbidities	Yes	No
ESRD	24 (68.6%)	11 (31.4%)
CKD	11 (31.4%)	24 (68.6%)
Diabetes	18 (51.4%)	17 (48.6%)
Hypertension	21 (60%)	14 (40%)

**Table-II:** Outcomes of AVF among the patients admitted for AVF

Outcomes of AVF	Yes	No
Thrill at the of AVF	27 (77.1%)	8 (22.9%)
Backflow at cut end of cephalic vein	26 (74.3%)	9 (25.7%)
Pulse at the site of fistula	34 (97.1%)	1 (2.9%)
Surgeon satisfied	26 (74.3%)	9 (25.7%)

**Fig.2:**
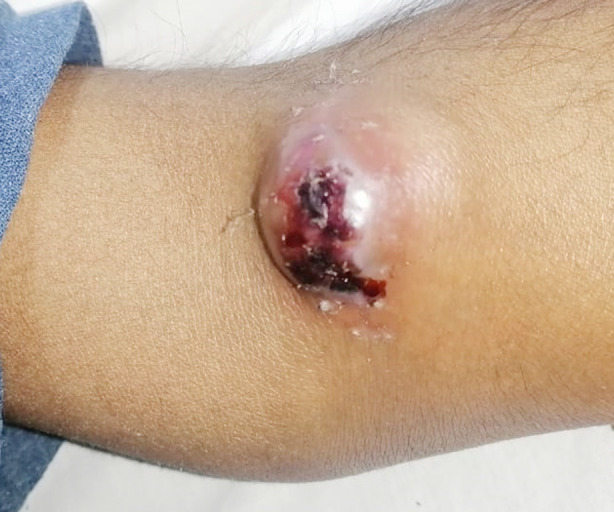
Pseudo aneurysm secondary to AVF.

The chi-square analysis highlighted that among the patients when thrill at the site of AVF and backflow at cut end of cephalic vein were present the surgeons were satisfied, and the higher satisfaction among the surgeon was achieved in the AVF created at brachiocephalic artery. The t-test analysis didn’t reveal significant findings and showed that the diameter of vessels doesn’t contribute in surgeon satisfaction (Table-IV). In current study we observed that one patient developed pseudo aneurysm secondary to AVF, the patient was shifted to dressing room for pressure dressing. The pseudo aneurysm secondary to AVF is depicted in Fig.-2.

**Table-III:** Association of surgeon satisfaction outcomes and site of AVF.

Outcomes of AVF		Surgeon satisfied		

		Yes	No	
Thrill at the site of AVF	Yes (n=27)	26 (96.3%)	1 (3.7%)	0.001*
	No (n=8)	0	8 (100%%)	
Backflow at cut end of cephalic vein	Yes (n=26)	23 (88.5%)	3 (11.5)	0.003*
	No (n=9)	3 (33.3)	6 (66.7%)	
Pulse at the site of fistula	Yes (n=34)	26 (76.5%)	8 (23.5%)	0.257
	No (n=1)	0	1 (100%)	
Site of AVF				
Brachiocephalic (n=21)		18 (85.7%)	3 (14.3%)	0.068
Radiocephalic (n=14)		8 (57.1%)	6 (42.9%)	

Significant p-value.

**Table-IV:** Comparison of diameter of vessels with surgeon satisfaction after AVF.

Diameter of vessels	Surgeon satisfied	N=	Mean	Std. Deviation	p-value
Post anastomosis diameter of fistula	Yes	26	3.1846	2.27674	0.246
	No	9	2.1611	2.11539	
Caliber of Cephalic Vein At wrist	Yes	26	1.677	.6501	0.920
	No	9	1.700	.3354	
Caliber of Cephalic Vein At cubital fossa	Yes	26	2.673	.7063	0.466
	No	9	2.933	1.3702	

## DISCUSSION

Among the factors that leads to AVF maturation failure surgical and suturing techniques have been considered as a major predisposing factor by surgeons. Various studies have reported advantages and disadvantages of continuous and interrupted suturing techniques.^7^,^8^ Regarding the continuous suturing technique, it has been documented that this technique for AV anastomosis contributes to aberrant hemodynamics in the anastomotic area because it can cause uneven suture line tension and a purse-string effect, which can cause anastomotic constriction and sub-optimal anastomotic compliance and predispose to AVF failure.^9^,^10^ However, when compared to continuous suturing techniques interrupted suturing technique have been preferably used by the surgeons to avoid the disadvantages related to continuous suturing techniques.^11^ But to further endorse its benefits assessment of the outcomes of interrupted technique and surgeon satisfaction is of prime importance hence the study was aimed to document the Outcomes of AV fistula formation for dialysis patients using interrupted sutures.

In the current study 35 patients were evaluated to assess the outcomes of the AVF. Surgeon satisfaction rate was seemed to be 26 (74.3%). The mean age of participants was 59.34 ± 15.48, which is similar to the other studies conducted and these are correlated with the age of onset of ESRD however, CKD does not follow the same findings regarding the age.^12^,^13^ In the current study it was observed that comorbidities such as diabetes and hypertension does not lead to FM, neither these affected the surgeon satisfaction hence, in accordance with the reported findings of Siddiqui MA et al., these chronic diseases do not interfere in the maturation of AVF directly however, uncontrolled diabetes may increase the chances of infection and persistent hypertension may also lead to FM therefore, managing theses chronic illnesses during and after surgery may lead to good outcomes of AVF in hemodialysis patients.^14^-^16^

Furthermore, to identify the AVF outcomes and surgeon satisfaction thrill at the site of AVF, backflow at cut end of cephalic vein and pulse at the site of AVF were recorded. The observation revealed that presence of the thrill at the site of AVF and backflow at cut end of cephalic vein were related to surgeon satisfaction rate. In patients where both of these factors were absent surgeon marked it as unsatisfactory and ultimately the AVF become nonfunctional in follow-up visits. It has been documented that in addition to the interference of the uremic condition, coagulation, and inflammation, the development of stenosis and thromboses can be seen as the result of a triangular interaction between (1) the biomaterial used, (2) flow and blood properties such as shear rate and stress, flow rates oscillations, and backflow, and (3) the geometrical shape of vessels and grafts regarding, that is, outer and inner diameter, length, and curvature in relation to anticoagulation conditions all to gather these factor may predispose a patient to poor outcomes of an VF.^17^ In this regard, the absence of thrill immediately or at 24 hours have been corelated with the poor outcomes of AVF.^18^ Presence of thrill has been associated with the good blood at the site of AVF and its absence marks the stenosis that predisposes the patient to poor out outcome of AVF.^19^

There was no significant relationship of anatomical variations in the current study the mean diameter of the vessels remain insignificant in both the patients for which surgeon was satisfied or not. Contrary to this Kordzadeh A et al. has demonstrated that if the cutoff of > 1.5 mm in the cephalic vein and 1.6 mm in the radial artery diameters met there are greater chances of failure to mature which may ultimately contribute to the primary failure of the AVF.^10^

### Limitations:

It includes small sample size and some patients were lost to long term follow-ups.

## CONCLUSION

The findings of current study highlight that presence of thrill at the site of AVF and backflow at cut end of cephalic vein have strong association with good prognosis of AVF. Furthermore, diabetes and hypertension does not affect the prognosis of AVF neither it is related to diameter of vessels.

### Authors’ Contribution:

**FN** conceived, designed, and did statistical analysis & editing of manuscript, and is responsible for the integrity of research.

**RHJ** designed, did acquisition and revised critically.

**SN** and **HK** did data collection and manuscript writing

**FN, RHJ, SN, and HK** gave final approval of the version to be submitted and agreed to be ac-countable for all aspects of the work.
